# Assessing concordance between *Campylobacter* prevalence in broilers and human cases before and during the COVID-19 pandemic in Lower Saxony, Germany, considering fresh chicken meat consumption patterns

**DOI:** 10.3389/fvets.2024.1392677

**Published:** 2024-05-09

**Authors:** Tobias Nolte, Fabian Spieß, Anne-Katrin Jacobs, Nicole Kemper, Christian Visscher

**Affiliations:** ^1^Science and Innovation for Sustainable Poultry Production (WING), University of Veterinary Medicine Hannover, Foundation, Vechta, Germany; ^2^Institute for Animal Nutrition, University of Veterinary Medicine Hannover, Foundation, Hannover, Germany; ^3^Institute for Animal Hygiene, Animal Welfare and Farm Animal Behaviour, University of Veterinary Medicine Hannover, Foundation, Hannover, Germany

**Keywords:** foodborne pathogen, One Health, campylobacteriosis, poultry, fresh chicken meat, COVID-19 pandemic

## Abstract

As the most common foodborne disease, number of campylobacteriosis decreased in Germany with the beginning of the COVID-19 pandemic in 2020. As the consumption of fresh chicken meat is a major risk factor for human infection, this study investigated the relationship between *Campylobacter* contamination levels on chicken carcasses and human cases in Lower Saxony, Germany and observed fresh chicken meat consumption patterns between 2018 and 2021 including the time of the COVID-19 pandemic. *Campylobacter* levels in broilers and human cases were classified based on the median and descriptively analysed per week using contingency tables. Before the COVID-19 pandemic (2018 and 2019), high *Campylobacter* contamination levels on neck samples and many human cases were more present, whereas with the beginning of the COVID-19 pandemic (2020 and 2021), low contamination levels on chicken carcasses and few human cases were more present. Lowest concordance between both parameters was shown in 2018 (Cohen’s cappa coefficient: 0.37) and 2020 (0.38). The highest concordance was examined in 2021 (0.69). The private consumption of fresh chicken meat in Lower Saxony increased significantly with the beginning of the COVID-19 pandemic in 2020 by 63.9 tonnes compared to 2019 to an average of 453.5 tonnes per week. Public health measures and a reduced number of medical treatments have undoubtedly had an impact on less reported human cases during the COVID-19 pandemic. However, number of human cases remained at a low level in Germany in 2023 while chicken meat consumption increased. Thus, further risk assessments regarding the risk of campyloabcteriosis due to chicken meat consumption should include the country of origin, as the level of contamination of chicken carcasses varies between European countries.

## 1 Introduction

*Campylobacter* spp. is the most common foodborne bacterial pathogen in Germany and the European Union (1, 2). The consumption of chicken meat has been identified as an important risk factor for campylobacteriosis in several countries (3–6). Around one third of human cases could be avoided if the risk factor of eating chicken was not present (7). Chickens as a reservoir for *Campylobacter* can attribute for 50 to 80% of human cases (8). Controlling campylobacteriosis as a zoonotic pathogen is part of the One Health Approach, which states that human and animal health and the environment are closely interlinked. With the introduction of mandatory testing for *Campylobacter* at slaughterhouses as part of the process hygiene criterion (PHC) for *Campylobacter* to reduce the risk of campylobacteriosis, the One Health approach has become increasingly important along the chicken meat production value chain in recent years (9). An evaluation of neck skin samples of chicken carcasses from several slaughter lines in Northwest Germany since the beginning of microbiological testing for *Campylobacter* in January 2018 showed, that the proportion of highly contaminated samples (>1,000 colony forming units per gram (cfu/g)) of *Campylobacter* dropped by almost half from 19.4% in 2018 to 10.5% in 2021 (10).

The *per capita* consumption of chicken meat in Germany increased by 1 kg from 14.3 kg in 2018 to 15.3 kg in 2021 (11). Thus, chicken meat plays an increasingly important role for nutritional reasons and the poultry sector is therefore of growing economic importance. In Germany, 486,331 tonnes of poultry meat were purchased at retail in 2020 (12). This was 14.6% more than the previous year and can be related to the temporary closure of restaurants at certain times during the COVID-19 pandemic. Panzone et al. (13) showed that the COVID-19 restrictions in the UK resulted in an increase in sales for food retailers and a simultaneous loss in sales for restaurants and non-food retailers (13).

The number of notifiable gastrointestinal diseases decreased in Germany with the beginning of the COVID-19 pandemic in 2020. Regarding campylobacteriosis, only 46,000 human cases were reported in 2020, which was 33% less than the median of the five previous years (1). The number of *Campylobacter* enteritis cases decreased in many countries worldwide in 2020 (14). In the European Union, a total of about 120,000 cases of campylobacteriosis were reported in 2020 (15). Previous studies have shown seasonal fluctuations with a higher number of human cases in the summer months (16, 17). Higher prevalences of *Campylobacter* in the summer months were also detected in broiler flocks (18, 19). Jore et al. (20) described a concordant seasonality between *Campylobacter* in broilers and human *Campylobacter* enteritis cases in six European countries (20). Thus, there seems to be a relationship between *Campylobacter* in broilers and the number of campylobacteriosis. Correlation analysis showed a higher association between highly contaminated (>1,000 cfu/g *Campylobacter*) neck samples of chicken carcasses and *Campylobacter* enteritis cases in Northwest Germany before the COVID-19 pandemic than during the pandemic (10).

With the beginning of the COVID-19 pandemic, government at federal and state level revised strict public health measures to reduce the spread of the coronavirus. There are numerous hypotheses to what extent a decrease in gastrointestinal diseases like *Campylobacter* was related to the COVID-19 pandemic and the associated public health measures in Germany (21). These include fewer restaurant visits, the ban on major events, improved hand hygiene, less travel activities and reduced use of medical care (22–24). As restaurants were closed at certain times during the COVID-19 pandemic, the risk factor of *Campylobacter* infection due to the consumption of chicken meat at the restaurant should have been less relevant at that time.

This study analysed the relationship between *Campylobacter* contamination levels in broilers at slaughterhouses in Northwest Germany and the number of *Campylobacter* enteritis cases in Lower Saxony (LS), a federal state of Germany with a population of around 8 million people. The period of investigation covered 4 years from 2018 to 2021. The aim was to analyse to which extent *Campylobacter* contamination levels on chicken carcasses in slaughterhouses and the number of *Campylobacter* enteritis cases in LS are related. Taking into account the special situation of the COVID-19 pandemic, the aim of the study was to understand the risk associated with the private consumption of fresh chicken meat during the pandemic. With *Campylobacter* being the most important zoonotic pathogen regarding foodborne diarrhoeal diseases, the scientific results also serve the One Health approach in Germany.

## 2 Materials and methods

### 2.1 *Campylobacter* levels in broilers

As part of the PHC for *Campylobacter*, slaughterhouses in the European Union have been obliged to test neck skin samples of chicken carcasses for *Campylobacter* since 1 January 2018. This study included the *Campylobacter* contamination levels on chicken carcasses, obtained from the food business operators as part of the PHC in accordance with Regulation (EC) No 2017/1495, from several slaughterhouses in Northwest Germany from 2018 to 2021 (9). A total of 15 neck skin samples were analysed weekly. Three samples were pooled (one pool sample weighing 26 g) and examined in the laboratory using the analytical reference method ISO 10272-2 (25). Thus, 9 portions (234 mL) of buffered peptone water were added to the 26 g test quantity for the initial dilution in the laboratory. 10 mL of the mixture of the initial dilution were added to an empty sterile tube; 1 mL of this 10 mL were used for the enumeration of *Campylobacter* on selective plates. Each slaughterhouse had five weekly quantitative microbiological results on the occurrence of *Campylobacter* available since 1 January 2018.

### 2.2 Data on human *Campylobacter* cases

According to (§7) of the German Protection against Infection Act, campylobacteriosis is a notifiable disease. The laboratory detection is reported to the Robert Koch Institute (RKI), the federal public health institute, via the health authorities (16). The number of *Campylobacter* enteritis cases are publicly accessible via SurvStat@RKI 2.0 (26). In this research project, the number of reported *Campylobacter* enteritis cases in LS, Germany from 2018 to 2021 were included.

### 2.3 Methodology

The neck skin samples of chicken carcasses according to PHC for *Campylobacter* were valued based on the slaughter quantity of the individual slaughter lines. The geometric mean of all microbiological results (cfu/g *Campylobacter*) from all slaughter lines per calendar week (CW) was calculated. The median was determined using the geometric mean values from the first CW of 2018 to the 52nd CW of 2021. The median of 176 cfu/g *Campylobacter* was used to classify the contamination levels of chicken carcass neck samples into “low level of contamination” and “high level of contamination.”

A similar classification was used for the weekly reported human *Campylobacter* enteritis cases in LS. The median of reported *Campylobacter* enteritis cases from the first CW of 2018 to the 52nd CW of 2021 was determined (88 human cases) and used to classify the amount of cases into “few human cases” and “many human cases.”

For further analysis, a two-week delay was calculated between the examination of chicken carcasses at slaughterhouses and human cases, as has already been done in previous studies (27, 28).

### 2.4 Private consumption behavior of fresh chicken meat in Lower Saxony

The Association for Consumer Research (Gesellschaft für Konsumforschung (GfK)) is a consumer panel with a representative sample of consumers whose (purchasing) behavior is measured. This study considered the weekly private consumption behavior of fresh chicken meat in tonnes in LS from 1 January 2018 to 31 December 2021, in order to determine differences in the quantity of the private consumption behavior of fresh chicken meat. The average weekly private consumption behavior of fresh chicken meat in tonnes in LS was compared annually from 2018 to 2021. In addition, data were compared between specific time periods within a year. A time period extended over four CWs. A calendar year began with time period one from week 1–4, followed by time period two from week 5–8, etc., and the calendar year ended with time period 13 from week 49–52. This amounted to 13 time periods per calendar year.

### 2.5 Treatment cases in Lower Saxony specific to specialist groups

The Association of Statutory Health Insurance Physicians of LS (Kassenärztliche Vereinigung Niedersachsen (KVN)) provided the total number of human medical treatments for all specialist groups in LS. The number of medical treatments is available to us on a quarterly basis and is presented on a comparative basis from the first quarter of 2018 to the fourth quarter of 2021.

### 2.6 Statistical analysis

Data analysis was performed using the SAS statistical software package, version 7.1 (SAS Inst., Cary, NC, United States). After classification of *Campylobacter* contamination levels of chicken carcass neck samples and human cases, both characteristics were shown and descriptively analysed using table analyses. Pearson’s chi-square homogeneity test was used to determine if there was an association between the two parameters. Cohen’s kappa coefficient as a descriptive measure of agreement was analysed and evaluated according to Altman: values of <0.20 are poor, 0.21–0.40 fair, 0.41–0.60 moderate, 0.61–0.80 good and 0.81–1.00 very good (29). In addition, the McNemar test was used for differences in the marginal distributions.

The data on private consumption behavior of fresh chicken meat were analysed descriptively by mean values, minimum, maximum and standard deviation. To test for normal distribution, a Shapiro–Wilk test was performed. Data were checked for significant differences with the Ryan-Einot-Gabriel-Welsch-test (one-way ANOVA). All statements of statistical significance were based on *p* < 0.05.

## 3 Results

### 3.1 Relationship between *Campylobacter* levels in broilers and human *Campylobacter* enteritis cases

In the following section, each table (Tables 1–4) shows the relationship between *Campylobacter* levels on neck samples of chicken carcasses at slaughterhouse and human cases in LS for 1 year from 2018 to 2021. *Campylobacter* contamination levels on neck samples were classified into “low level of contamination” and “high level of contamination.” Human cases were also classified into “few cases” and “many cases.” Each table (Tables 1–4) illustrates 1 year (52 CWs) from 2018 to 2021.

**Table 1:** Relationship between *Campylobacter* contamination levels on chicken carcasses and human *Campylobacter* enteritis cases in Lower Saxony in 52 calendar weeks in 2018.

2018			
	Neck samples		
Human cases	Low level of contamination	High level of contamination	Total
*Few human cases*	12	4	16
*Many human cases*	12	24	36
*Total*	24	28	52

Pearson chi-square test: 7.74 (*p* < 0.05). *Campylobacter* levels (colony-forming units per gram) on chicken carcasses were classified into “low level of contamination” and “high level of contamination” using the median of all microbiological results from 2018 to 2021. The same method was used for weekly reported human *Campylobacter* enteritis cases in Lower Saxony, classified into “few human cases” and “many human cases.”

**Table 2:** Relationship between *Campylobacter* contamination levels on chicken carcasses and human *Campylobacter* enteritis cases in Lower Saxony in 52 calendar weeks in 2019.

2019			
	Neck samples		
Human cases	Low level of contamination	High level of contamination	Total
Few human cases	18	5	23
Many human cases	8	21	29
Total	26	26	52

Pearson chi-square test: 13.18 (*p* < 0.05). *Campylobacter* levels (colony-forming units per gram) on chicken carcasses were classified into “low level of contamination” and “high level of contamination” using the median of all microbiological results from 2018 to 2021. The same method was used for weekly reported human *Campylobacter* enteritis cases in Lower Saxony, classified into “few human cases” and “many human cases.”

**Table 3:** Relationship between *Campylobacter* contamination levels on chicken carcasses and human *Campylobacter* enteritis cases in Lower Saxony in 52 calendar weeks in 2020.

2020			
	Neck samples		
Human cases	Low level of contamination	High level of contamination	Total
Few human cases	22	12	34
Many human cases	4	14	18
Total	26	26	52

Pearson chi-square test: 8.5 (*p* < 0.05). *Campylobacter* levels (colony-forming units per gram) on chicken carcasses were classified into “low level of contamination” and “high level of contamination” using the median of all microbiological results from 2018 to 2021. The same method was used for weekly reported human *Campylobacter* enteritis cases in Lower Saxony, classified into “few human cases” and “many human cases.”

**Table 4:** Relationship between *Campylobacter* contamination levels on chicken carcasses and human *Campylobacter* enteritis cases in Lower Saxony in 52 calendar weeks in 2021.

2021			
	Neck samples		
Human cases	Low level of contamination	High level of contamination	Total
Few human cases	24	6	30
Many Human cases	3	19	22
Total	27	25	52

Pearson chi-square test: 24.81 (*p* < 0.05). *Campylobacter* levels (colony-forming units per gram) on chicken carcasses were classified into “low level of contamination” and “high level of contamination” using the median of all microbiological results from 2018 to 2021. The same method was used for weekly reported human *Campylobacter* enteritis cases in Lower Saxony, classified into “few human cases” and “many human cases.”

As shown in Table 1, most of the 52 CWs were located in the lower right quadrant in 2018. Thus, a “high level of contamination” on neck samples and “many human cases” occurred at the same time in 24 CWs. The smallest number was located in the upper right quadrant, 4 weeks in which “few human cases” and a “high level of contamination” on neck samples occurred at the same time. In 2018, “many human cases” were reported in a total of 36 CWs, whereas “few human cases” were reported in 16 CWs.

Table 2 shows the highest number in the lower right quadrant, 21 CWs in which “high level of contamination” on neck samples and “many human cases” occurred at the same time in 2019. In 18 CWs, a “low level of contamination” on neck samples and “few human cases” occurred simultaneously. The smallest number (five CWs) is shown in the upper right quadrant with a “high level of contamination” on neck samples and “few human cases” reported at the same time. “Few human cases” were reported in 23 CWs, “many human cases” in 29 CWs.

In 22 CWs, there were a “low level of contamination” on neck samples and “few human cases” at the same time in 2020 as shown in Table 3. A “high level of contamination” and “many human cases” were reported in 14 CWs. The lowest amount (4 CWs) is located in the lower left quadrant in 2020 with a “low level of contamination” and “many human cases” reported at the same time. In 2020, “few human cases” were reported in 34 CWs and “many human cases” in 18 CWs.

As shown in Table 4, most 24 CWs were located in the upper left quadrant in which a “low level of contamination” on chicken carcass neck samples and “few human cases” occurred at the same time. In 19 CWs, a “high level of contamination” and “many human cases” were reported simultaneously. The lowest amount of 3 CWs was located in the lower left quadrant in 2021 in which a “low level of contamination” on neck samples and “many human cases” occurred at the same time. “Few human cases” were reported in 30 CWs and “many human cases” in 22 CWs.

Table 5 shows the statistical results of Cohen’s kappa coefficient and the McNemar test for each contingency table (Tables 1–4) from 2018 to 2021. The lowest Cohen’s kappa-coefficient value of 0.37 was obtained in 2018 and the highest value in 2021 (0.69). In 2020, the Cohen’s kappa-coefficient value of 0.38 was almost as low compared to 2018. In 2019, there was a Cohen’s kappa-coefficient value of 0.5. McNemar test with a value of 4 showed that there was a higher difference in the marginal distributions in 2018 and 2020 compared to 2019 and 2021. However, the *p*-value of the McNemar test was not significant, neither in 2018 nor in 2020.

**Table 5:** Cohen’s kappa coefficient, McNemar test and exact *p*-value of McNemar test for contingency tables (Tables 1–4) from 2018 to 2021.

Statistical test	2018	2019	2020	2021
Cohen’s kappa coefficient:	0.37	0.5	0.38	0.69
McNemar test:	4	0.69	4	0.5
Exact *p*-value to McNemar test:	0.08	0.58	0.08	0.73

Table 6 shows the development of the following parameters from 2018 to 2021: Proportion (%) of CWs with ‘high’ *Campylobacter* contamination levels on chicken carcasses, proportion (%) of CWs with ‘many’ human *Campylobacter* enteritis cases, and the average weekly private consumption behavior of fresh chicken meat (tonnes) in Lower Saxony. The percentage of CWs with high *Campylobacter* contamination levels on chicken carcasses was highest in 2018 (53.8%) and lowest in 2021 (48.1%). In 69.2% of the CWs in 2018, many human cases were reported. In 2020, many human cases were reported in only 34.6% of the CWs. The average weekly private consumption of fresh chicken meat was highest in 2020. The lowest amount was consumed in 2018. Statistical analyses of this parameter are shown in Table 7.

**Table 6:** Overview of the development of the following parameters: proportion (%) of calendar weeks with ‘high’ *Campylobacter* contamination levels on chicken carcasses, proportion (%) of calendar weeks with ‘many’ human *Campylobacter* enteritis cases, and the average weekly private consumption behavior of fresh chicken meat (tonnes) in Lower Saxony from 2018 to 2021.

Measure	2018	2019	2020	2021
CW (%) with high level of contamination on chicken carcasses:	53.8	50.0	50.0	48.1
CW (%) with many human cases:	69.2	55.8	34.6	42.3
Average weekly private consumption behavior of fresh chicken meat (tonnes):	352.6	389.5	453.4	433.9

**Table 7:** Average weekly private consumption behavior of fresh chicken meat in tonnes in Lower Saxony from 2018 to 2021 according to Gesellschaft für Konsumforschung (GfK).

Year	Chicken meat (tonnes)
2018	352.6^A^ ± 93.0
2019	389.5^A^ ± 88.1
2020	453.4^B^ ± 82.9
2021	433.9^B^ ± 86.5

^A,B^Means in a column with different superscripts differ significantly (*p* < 0.05).

### 3.2 Private consumption behavior of fresh chicken meat in LS

In the following section, the private consumption behavior of fresh chicken meat in tonnes in LS from 2018 to 2021 is presented.

#### 3.2.1 Private consumption behavior of fresh chicken meat per year

Table 7 displays and compares the average weekly private consumption behavior of fresh chicken meat in tonnes in LS from 2018 to 2021. There was a significantly higher amount of fresh chicken meat consumed in 2020 and 2021 compared to 2018 and 2019 (*p* < 0.05) with the highest amount consumed in 2020 with 453.4 tonnes. The lowest amount of fresh chicken meat was consumed in 2018 (352.6 tonnes), this was about 100 tonnes less compared to 2020.

#### 3.2.2 Private consumption behavior of fresh chicken meat per time period

**Figure 1:**
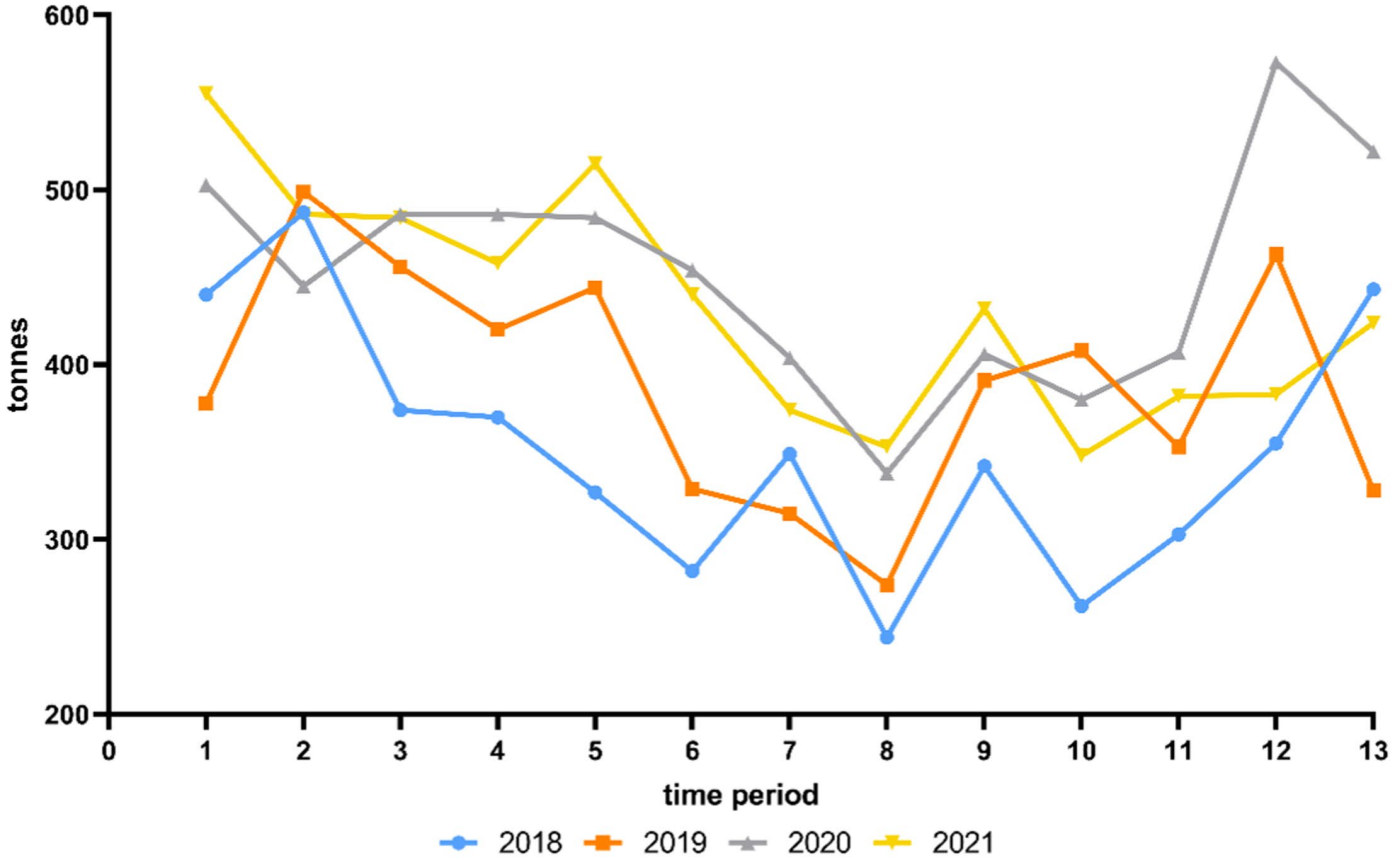
Average weekly private consumption behavior of fresh chicken meat in tonnes in Lower Saxony for each time period from 2018 to 2021 according to Gesellschaft für Konsumforschung (GfK).

Figure 1 shows the average weekly private consumption behavior of fresh chicken meat in tonnes in LS for each time period from 2018 to 2021. Additional statistical analyses are shown in Supplementary Table A.

As shown in Figure 1, in 2018 and 2019 the private consumption behavior of fresh chicken meat was significantly higher in time period 2 (Supplementary Table A). In 2020, a significantly higher amount was consumed in time period 12 and in 2021, the consumption was significantly higher in time period 1 (Supplementary Table A). Over the 4 years from 2018 to 2021, a significantly lower proportion was consumed in time period 8 in each year (*p* < 0.05) (Supplementary Table A).

### 3.3 Medical treatments in Lower Saxony

**Figure 2:**
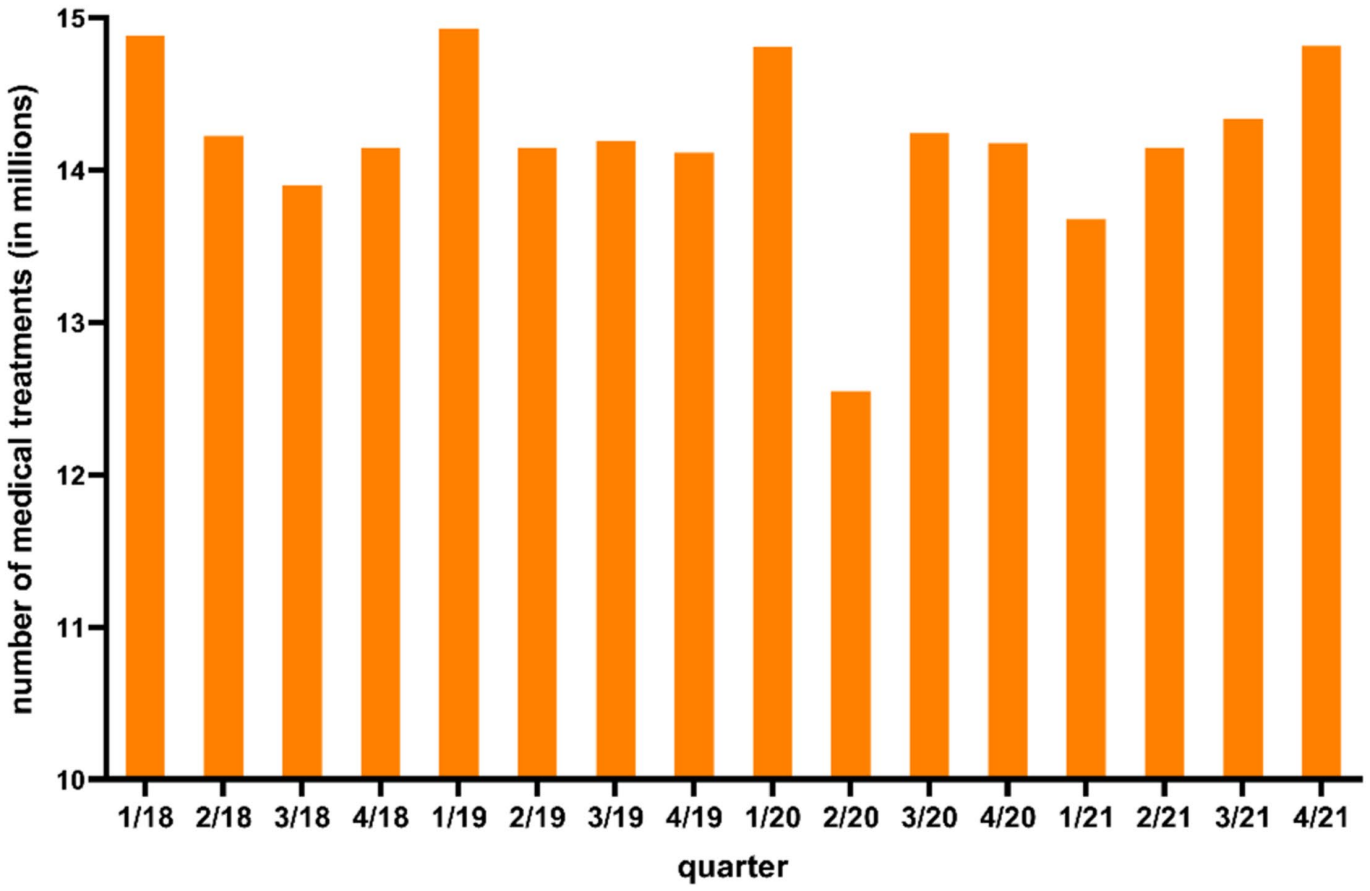
Number of total human medical treatments in Lower Saxony from first quarter 2018 to fourth quarter 2021 according to Kassenärztliche Vereinigung Niedersachsen (KVN).

Figure 2 shows the number of human medical treatments in LS provided by the Association of Statutory Health Insurance Physicians of LS (KVN) from the first quarter of 2018 to the fourth quarter of 2021.

As shown in Figure 2, the number of medical treatments were lower in the second quarter 2020 (12.5 million medical treatments) compared to the other quarters.

## 4 Discussion

### 4.1 Relationship between *Campylobacter* levels in broilers and human *Campylobacter* enteritis cases

The temporal relationship between *Campylobacter* contamination levels in broilers in slaughterhouses and human cases in LS was analysed from 2018 to 2021. A delay of 2 weeks between microbiological testing of chicken carcass neck samples and human cases in LS was calculated based on an expected time lag between slaughter and consumption up to the laboratory diagnostic detection of campylobacteriosis. A two-week delay has been adopted in previous studies (27, 28).

During the investigated period, which included the COVID-19 pandemic, the highest concordance between contamination levels on neck samples and reported *Campylobacter* enteritis cases in LS was analysed in 2021 (Cohen’s Kappa Coefficient: 0.69) (Table 5). A lower concordance was examined in 2018 (0.37) and 2020 (0.38). A concordant seasonality of broiler prevalence and human cases has been shown before in the literature (20). Concerning the relationship between *Campylobacter* in broilers and human cases, an infection of poultry and humans by a common source has been discussed previously (30, 31). Thus, climatic factors such as temperature and humidity were considered as a source of human incidence (32, 33).

This study showed that before the COVID-19 pandemic, in 2018 and 2019, in the majority of the 52 CWs a high *Campylobacter* contamination level on neck samples and many human cases were recorded at the same time. However, with the beginning of the COVID-19 pandemic in 2020, in most of the CWs a low level of contamination on chicken carcasses and few human cases were present simultaneously in 2020 and 2021. These results align with Rosenquist et al. (34) who identified an association between a reduction in contamination levels of *Campylobacter* found on chicken carcasses in slaughterhouses and a reduction in *Campylobacter* enteritis cases. The closing of restaurants at various times during the COVID-19 pandemic might have led to fewer reported human cases, as eating at restaurants poses a high risk of campylobacteriosis (7). To analyse this risk factor during the COVID-19 pandemic, additional data on the proportion of chicken meat consumed at restaurants would be necessary. However, there is no data acquisition from the state or private sector on the amount of chicken meat consumed in the hospitality industry in LS. With eating at restaurants being a risk factor for campylobacteriosis, the country of origin of chicken meat consumed at restaurants is of great interest, as *Campylobacter* contamination levels on chicken carcasses in slaughterhouses, examined in frame of the PHC for *Campylobacter*, vary between European countries (2, 35). Depending on whether chicken meat originated from German farms or abroad with possibly higher *Campylobacter* contamination levels on chicken carcasses, this could have influenced the risk of campylobacteriosis due to chicken meat consumed at German restaurants. Tedersoo et al. (36) investigated a higher risk of campylobacteriosis by imported fresh chicken meat compared to chicken meat originating from Estonia. Low *Campylobacter* contamination levels on chicken carcasses at domestic abattoirs, as previously described in Northwest Germany, would therefore have had an impact on less reported human cases during the COVID-19 pandemic (10). Skarp et al. (31) mentioned that in countries with low *Campylobacter* levels in broilers, other sources than broilers may play a larger role for human infection.

Other risk factors for campylobacteriosis might have also been less relevant during the COVID-19 pandemic, despite the risk factor of chicken meat consumption. Regarding the discordant values (Tables 1–4) before the pandemic, in 2018 and 2019, low contamination levels on chicken carcasses and many human cases were reported at the same time in the majority of the weeks. During the pandemic (2020 and 2021), those values shifted and high contamination levels on chicken carcasses and few human cases were reported simultaneously in the majority of the weeks. Especially in 2018 and 2020 the asymmetry was high (*p*-value of McNemar test in both years: 0.08) (Table 5). In 2018 in particular, a low level of *Campylobacter* on chicken carcasses and many human cases were reported in 12 CWs as shown in Table 1, whereas in 2020, high *Campylobacter* levels on chicken carcasses and few human cases were reported in the majority of the 12 CWs as shown in Table 3. These results suggest that before the COVID-19 pandemic in 2018 and 2019 external risk factors which are not related to the consumption of chicken meat, such as travelling abroad, may have contributed more to the number of *Campylobacter* enteritis cases than during the COVID-19 pandemic (37). Since foreign travel in Germany decreased in 2020 due to the pandemic compared to the previous year (38). In contrast, with the beginning of the COVID-19 pandemic, public health measures like social distancing and handwashing, and a lower number of visits to the physicians may have led to a reduced number of human cases despite the simultaneously high *Campylobacter* contamination level on chicken carcasses at that time (Tables 3, 4).

With the beginning of the COVID-19 pandemic in Germany in March 2020, fewer *Campylobacter* enteritis cases were reported to the RKI in LS compared to previous years (Supplementary Table B). With the first measures enacted by the state government in CW 12 in 2020 to reduce the spread of the coronavirus, less than half as many human cases were reported in LS in the time period four from week 13–16 in 2020 (99 human cases) compared to the same period in 2019 (227 human cases) (39, 40) (Supplementary Table B). It can be assumed that the fewer reported human cases were partly attributed to a reduced utilisation of medical care with the beginning of the pandemic. As shown in Figure 2 the number of human medical treatments in LS were lower in the second quarter of 2020 compared to other quarters. Jördens et al. (41) reported a decrease in gastroenterology consultations in Germany in the second quarter of 2020 compared to 2019. Thus, during the COVID-19 pandemic *Campylobacter* enteritis cases and other infectious diseases were probably not detected as often as in previous quarters, especially during the second quarter of 2020 (Figure 2). It is likely that person-to-person transmitted diseases showed a stronger decrease compared to foodborne transmitted pathogens like *Campylobacter* because of public health measures like social distancing and hand washing (42–44). A direct risk assessment including the number of human medical treatments in LS could not be carried out, since the Association of Statutory Health Insurance Physicians of LS [Kassenärztliche Vereinigung Niedersachsen (KVN)] provided the data only on a quarterly basis. In addition, the number of human medical treatments in LS included all specialist groups, therefore a differentiation between specialist groups is not possible. The number of gastroenterology consultations would have been necessary for a differentiated risk assessment.

### 4.2 Private consumption behavior of fresh chicken meat in LS

*Campylobacter* incidence was probably affected by different public health restrictions to contain the spread of the coronavirus, which included the closure of restaurants at various times during the COVID-19 pandemic in LS. This primarily implicated the first hard lockdown starting in CW 12 in 2020 and the second partial lockdown starting in CW 45 in 2020 (39, 40, 45). This led to a massive decline in eating out consumption and a sharp drop in sales in the hospitality industry from March to June 2020 and from November 2020 to March 2021 as shown in Supplementary Figure B, with highest sales losses in April 2020 (−72.6%). Since gradual lifting of the measures in the hospitality sector was first decreed in May 2020, more precisely in CW 20 in 2020 and CW 21 in 2020 (46, 47), the continued high sales losses in LS of 38.8% in June 2020 compared to 2019 and an ongoing reduced mobility behavior in LS in June 2020 could be an indication that citizens were unsettled by the coronavirus and still avoided eating out (Supplementary Figures B,C)

The *per capita* consumption of chicken meat in Germany increased from 14.4 kg in 2019 to 14.9 kg in 2020 (11). The temporary closure of restaurants during the COVID-19 pandemic suggests that chicken meat consumption shifted from restaurants to private consumption at home. The average weekly private consumption behavior of fresh chicken meat in LS was significantly higher in 2020 and 2021 compared to 2018 and 2019, leading to the highest consumption behavior with an average of 453.4 tonnes per week in 2020 (Table 7). This was 63.9 tonnes per week more than in 2019, the year before the COVID-19 pandemic began. A particularly large amount of fresh chicken meat was consumed at the end of 2020 in time period 12 (573.5 tonnes per week) and time period 13 (522.5 tonnes per week) and at the beginning of 2021 with 555.6 tonnes per week in time period 1 (Figure 1). This in particular reflected the consequence of restaurants being closed at that time. There are previous studies that analysed a shift from out-of-home consumption towards food retailing at the time of the COVID-19 pandemic (48, 49). In particular, Weersink et al. (50) mentioned an increase of 50% in fresh chicken meat sold in grocery stores in Canada in mid-March 2020 compared to the same week in the previous year.

Fresh chicken meat from retail is often associated with an increased risk for campylobacteriosis due to a high prevalence of *Campylobacter* (51–53). Cross-contamination and poor hygienic behavior in the own kitchen pose additional risk factors for human incidence (54). It can therefore be assumed that the high amount of fresh chicken meat prepared at home during the COVID-19 pandemic in LS was accompanied by an increased risk of *Campylobacter* infection to consumers. However, the risk of contaminated retail chicken meat should have decreased in Germany in recent years due to lower *Campylobacter* contamination levels in slaughterhouses (10).

Contact reductions and restrictions in the event sector were further measures enacted by the government to reduce the spread of the coronavirus, with a potential influence on the reduced number of reported *Campylobacter* enteritis cases. As fewer major events and family celebrations such as weddings took place during the COVID-19, the risk factor of commercial food establishments experiencing campylobacteriosis was less prevalent at that time (37, 55). Social distancing may have also led to people having fewer barbecues so that the risk of *Campylobacter* transmission via barbecues was no longer present to the same extent as before the COVID-19 pandemic (5, 56–59).

## 5 Conclusion

The relationship between *Campylobacter* contamination levels on chicken carcass neck samples and reported human cases in LS was investigated between 2018 and 2021. The results show concordances between *Campylobacter* levels on chicken carcasses and human cases before and during the COVID-19 pandemic. The highest concordance was shown in 2021 (Cohen’s Kappa Coefficient: 0.69) and a lower concordance was shown in 2018 (0.37) and 2020 (0.38). The results of our study suggest that risk factors for campylobacteriosis, such as travelling abroad and eating at restaurants, had a different impact on the number of *Campylobacter* enteritis cases before and during the COVID-19 pandemic.

The private consumption behavior of fresh chicken meat in LS increased significantly in 2020 compared to the previous year by around 63.9 tonnes to an average of 453.5 tonnes per week, which is mainly due to the temporary closure of restaurants during the COVID-19 pandemic. A particularly high proportion of fresh chicken meat was consumed in LS at the end of 2020 and beginning of 2021. The highest consumption of 573.5 tonnes per week was recorded in time period 12 in 2020. As the consumption of fresh retail chicken meat is often associated with an increased risk of campylobacteriosis, this indicates that the risk was particularly high at that time (51–53). However, the risk should have decreased in recent years due to lower *Campylobacter* contamination levels in German slaughterhouses (10). Campylobacteriosis remains a challenge and must be pursued further as part of the One Health approach. A multifactorial approach is required to minimize *Campylobacter* as a pathogen at various stages along the chicken meat production value chain. This includes raising consumer awareness of campylobacteriosis as the most common foodborne pathogen in order to avoid *Campylobacter* infections and to reduce the number of human cases.

## Data availability statement

Data transfer and use agreements were made in order to receive and use the data from the food business operators. Requests to access the datasets should be directed to TN Tobias.Nolte@tiho-hannover.de.

## Author contributions

TN: Data curation, Formal analysis, Investigation, Methodology, Validation, Visualization, Writing – original draft. FS: Data curation, Formal analysis, Methodology, Visualization, Writing – review & editing. A-KJ: Funding acquisition, Writing – review & editing. NK: Conceptualization, Funding acquisition, Project administration, Supervision, Writing – review & editing. CV: Conceptualization, Data curation, Formal analysis, Funding acquisition, Investigation, Methodology, Project administration, Supervision, Validation, Visualization, Writing – review & editing.
