# Supplementary material for: Assessing concordance between Campylobacter prevalence in broilers and human cases before and during the COVID-19 pandemic in Lower Saxony, Germany, considering fresh chicken meat consumption patterns
Source: Front Vet Sci. 2024 May 9;11:1392677. doi: 10.3389/fvets.2024.1392677 (PMC11112064; doi:10.3389/fvets.2024.1392677)
Supplement: Supplementary file 1 [file Table_1.DOCX]

Supplementary Material

# Supplementary Data

Table A displays and statistically compares the average weekly private consumption behaviour of fresh chicken meat in tonnes in LS for each time period from 2018 to 2021.

**Table A.** Average weekly private consumption behaviour of fresh chicken meat in tonnes in Lower Saxony for each time period from 2018 to 2021 according to Gesellschaft für Konsumforschung (GfK).

| **Time period** | **2018** | **2019** | **2020** | **2021** |
| --- | --- | --- | --- | --- |
| 1 | 440.8 ^BC^ ± 35.2 | 378.3 ^ABC^ ± 72.2 | 503.1 ^BC^ ± 80.6 | 555.6 ^B^ ± 38.7 |
| 2 | 487.4 ^C^ ± 38.8 | 499.6 ^C^ ± 85.4 | 445.9 ^ABC^ ± 31.3 | 486.5 ^AB^ ± 62.4 |
| 3 | 374.2 ^ABC^ ± 23.0 | 456.9 ^BC^ ± 80.0 | 486.2 ^ABC^ ± 38.6 | 484.8 ^AB^ ± 28.6 |
| 4 | 370.2 ^ABC^ ± 134.4 | 420.4 ^ABC^ ± 34.7 | 486.9 ^ABC^ ± 99.8 | 458.7 ^AB^ ± 35.8 |
| 5 | 327.4 ^ABC^ ± 64.9 | 444.0 ^BC^ ± 43.5 | 484.3 ^ABC^ ± 33.0 | 515.7 ^AB^ ± 109.6 |
| 6 | 282.8 ^AB^ ± 61.7 | 329.0 ^AB^ ± 70.5 | 454.6 ^ABC^ ± 78.6 | 440.1 ^AB^ ± 63.8 |
| 7 | 349.2 ^ABC^ ± 112.8 | 315.8 ^AB^ ± 58.7 | 404.6 ^AB^ ± 50.2 | 374.2 ^A^ ± 19.9 |
| 8 | 244.6 ^A^ ± 60.7 | 274.1 ^A^ ± 54.3 | 338.1 ^A^ ± 42.0 | 353.9 ^A^ ± 15.6 |
| 9 | 342.1 ^ABC^ ± 21.6 | 391.7 ^ABC^ ± 39.7 | 406.3 ^AB^ ± 52.7 | 432.3 ^AB^ ± 121.5 |
| 10 | 262.1 ^A^ ± 56.9 | 408.2 ^ABC^ ± 71.4 | 380.4 ^AB^ ± 22.5 | 348.1 ^A^ ± 64.7 |
| 11 | 303.2 ^AB^ ± 38.4 | 353.4 ^ABC^ ± 114.9 | 407.7 ^AB^ ± 57.5 | 382.6 ^A^ ± 40.0 |
| 12 | 355.9 ^ABC^ ± 68.4 | 463.2 ^BC^ ± 70.2 | 573.5 ^C^ ± 93.3 | 383.6 ^A^ ± 36.2 |
| 13 | 443.6 ^BC^ ± 86.2 | 328.6 ^AB^ ± 42.5 | 522.5 ^BC^ ± 64.0 | 424.3 ^AB^ ± 119.3 |

A,B,C Means in a column with different superscripts differ significantly ( p < 0.05).

**Table B.** Number of *Campylobacter* enteritis cases in Lower Saxony for each time period from 2018 to 2021 according to SurvStat@RKI 2.0 (1). A time period extended over four calendar weeks. A calendar year began with time period 1 from week 1-4 and the calendar year ended with time period 13 from week 49-52.

| **Time period** | **2018** | **2019** | **2020** | **2021** |
| --- | --- | --- | --- | --- |
| 1 | 378 | 312 | 383 | 305 |
| 2 | 310 | 254 | 208 | 242 |
| 3 | 243 | 261 | 169 | 242 |
| 4 | 278 | 227 | 99 | 168 |
| 5 | 363 | 350 | 160 | 206 |
| 6 | 575 | 378 | 273 | 304 |
| 7 | 674 | 630 | 462 | 525 |
| 8 | 740 | 647 | 557 | 622 |
| 9 | 599 | 621 | 547 | 534 |
| 10 | 451 | 479 | 395 | 430 |
| 11 | 436 | 386 | 273 | 339 |
| 12 | 402 | 412 | 230 | 283 |
| 13 | 310 | 269 | 242 | 257 |

As illustrated in Table B, the highest number of human cases was reported in time period 8 in 2018 and the lowest number of cases was reported in time period 4 in 2020.

# Supplementary Figures and Tables

The Federal Statistical Office in Germany (Destatis) records and publishes the turnover figures in the hospitality industry for the different federal states in Germany (2). Figures A and B show the monthly turnover figures in the hospitality industry in LS from January 2018 to December 2021.


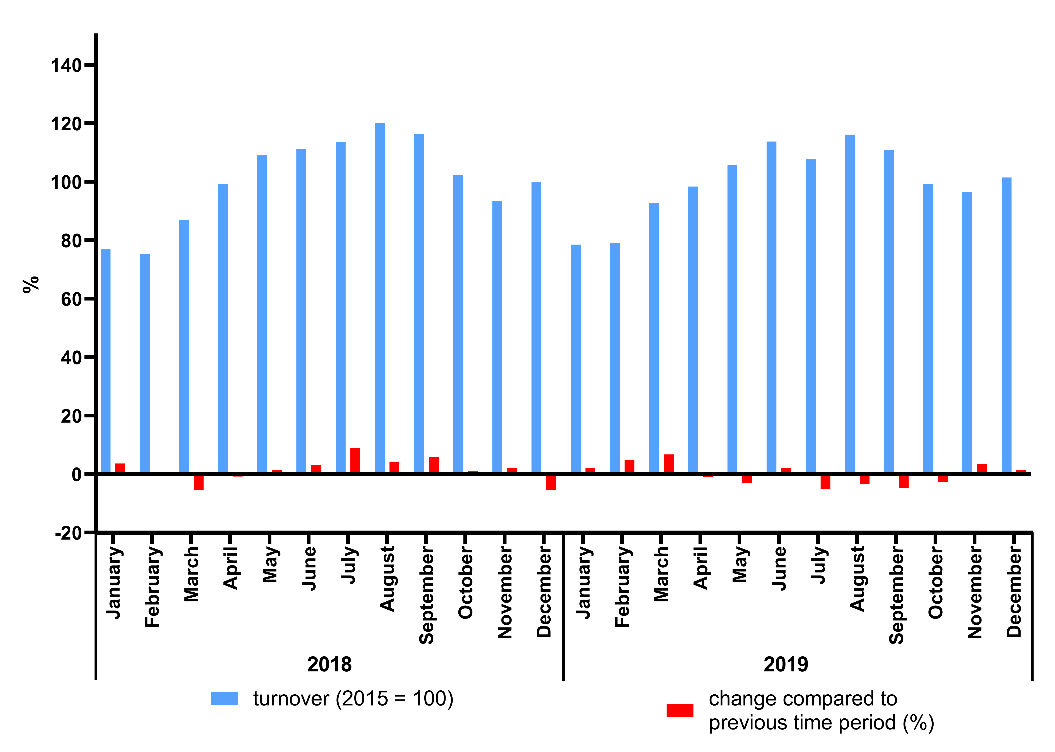


**Figure A.** Monthly turnover in the hospitality industry in Lower Saxony from January 2018 to December 2019 according to Destatis (2). The blue line shows the price-adjusted turnover com-pared to 2015. The red line shows the turnover compared to the same month in the previous year.


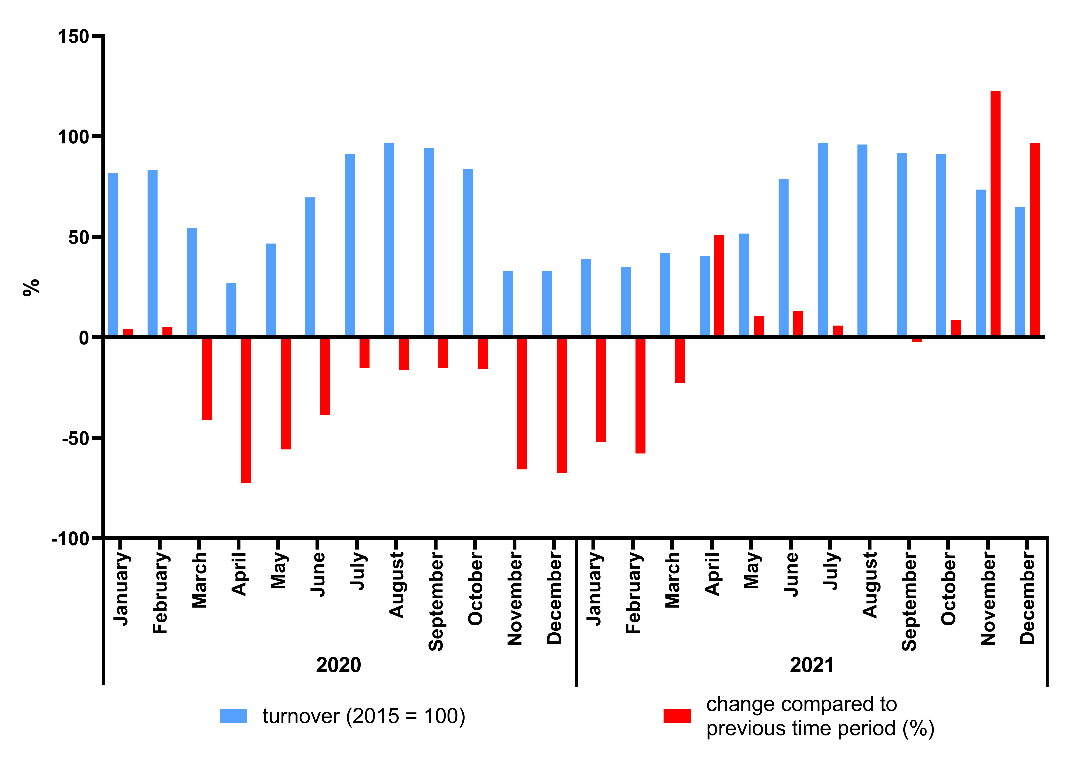


**Figure B.** Monthly turnover in the hospitality industry in Lower Saxony from January 2020 to December 2021 according to Destatis (2). The blue line shows the price-adjusted turnover com-pared to 2015. The red line shows the turnover compared to the same month of the previous year.

As shown in Figure B, the largest drop in turnover in 2020 was recorded in April with -72.6% compared to the same month of the previous year. In 2021, turnover dropped the most in February (-57.9%) compared to the same month in the previous year.

The Federal Statistical Office (Destatis) published mobility behaviour during the COVID-19 pandemic daily based on mobile phone data (3). The data were available for the various federal states in Germany. The daily mobility data in LS were added up and shown as an average value for different time periods in 2020 and 2021. A time period extended over four CWs. A calendar year began with time period 1 from week 1-4 and the calendar year ended with time period 13 from week 49-52, thus amounting to 13 time periods per calendar year. The values for the individual time periods in 2020 and 2021 illustrate the change in mobility pattern in LS compared to 2019.


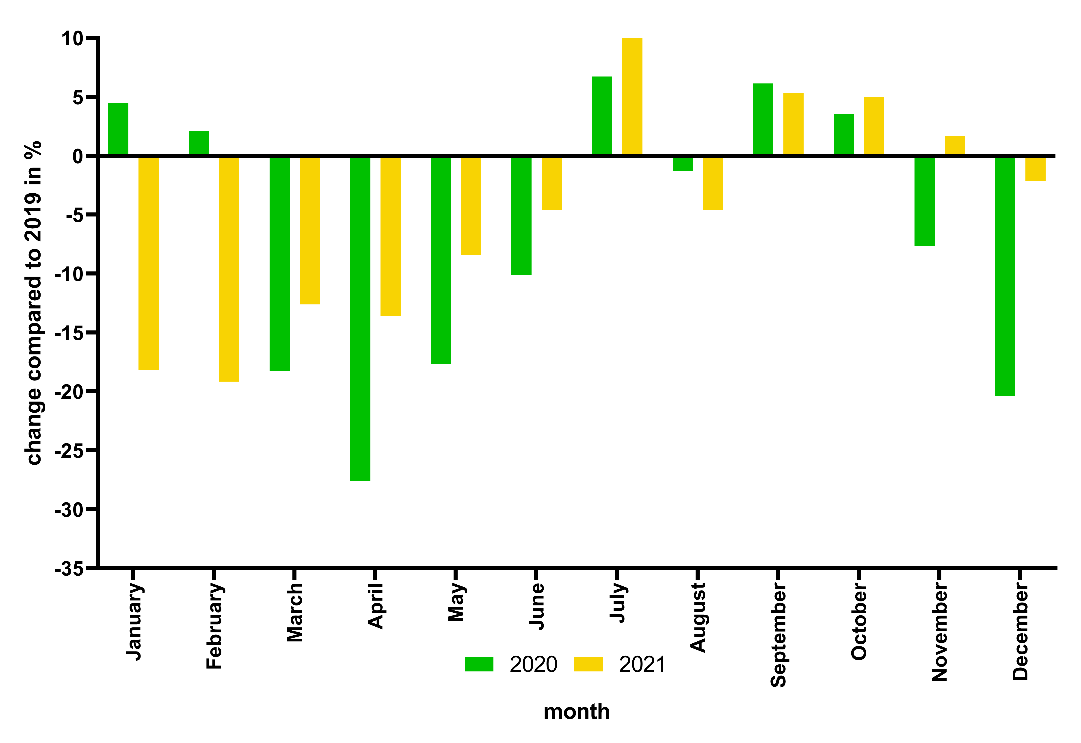


**Figure C.** Monthly changes in mobility behaviour in Lower Saxony in 2020 and 2021 compared to 2019 according to Destatis (3). The green line shows changes in mobility patterns for 2020 com-pared to 2019 in percent (%), the yellow line for 2021 compared to 2019 in percent (%) respectively.

As shown in Figure C, the strongest decline in mobility behaviour can be seen in April 2020.

1. Koch-Institut R. SurvStat@RKI 2.0 [Available from: <https://survstat.rki.de>.

2. (Destatis) SB. Die Datenbank des Statistischen Bundesamtes 2022 [Available from: <https://www-genesis.destatis.de/genesis/online>.

3. (Destatis) SB. Mobilitätsindikatoren auf Basis von Mobilfunkdaten 2022 [Available from: <https://www.destatis.de/DE/Service/EXSTAT/Datensaetze/mobilitaetsindikatoren-mobilfunkdaten.html>.
